# Effect of Exercise Training on Serum Transaminases in Patients With Nonalcoholic Fatty Liver Disease: A Systematic Review and Meta-Analysis

**DOI:** 10.3389/fphys.2022.894044

**Published:** 2022-06-28

**Authors:** Feng Hong, Yubo Liu, Veeranjaneya Reddy Lebaka, Arifullah Mohammed, Weibing Ye, Biqing Chen, Mallikarjuna Korivi

**Affiliations:** ^1^ Department of Sports Operation and Management, Jinhua Polytechnic, Jinhua, China; ^2^ Exercise and Metabolism Research Center, College of Physical Education and Health Sciences, Zhejiang Normal University, Jinhua, China; ^3^ Key Laboratory of Intelligent Education Technology and Application of Zhejiang Province, Zhejiang Normal University, Jinhua, China; ^4^ Department of Microbiology, Yogi Vemana University, Kadapa, India; ^5^ Faculty of Agro-Based Industry, Universiti Malaysia Kelantan, Kota Bharu, Malaysia

**Keywords:** physical actvity, fatty liver, older age group, NAFLD therapy, transaminase

## Abstract

**Background/Purpose:** Nonalcoholic fatty liver disease (NAFLD) constitutes a spectrum of liver diseases associated with various metabolic disorders. Exercise interventions reportedly manage the clinical outcomes of NAFLD, but their efficacy depends on exercise as well as characteristics of patient. We hypothesized that exercise could alleviate the elevated transaminases level, which may be associated with the characteristics of patients (age/bodyweight/sex) or exercise variables (frequency/intensity/duration). Therefore, we examined the effect of exercise on serum transaminases, and identified the variables influencing transaminases in NAFLD patients.

**Methods:** Article search was conducted using electronic databases (PubMed, Web of Science, EMBASE, ScienceDirect, Google Scholar) until December 2021. Studies that involved examination and comparison of the effect of an exercise intervention on alanine aminotransferase (ALT) and aspartate aminotransferase (AST) levels in NAFLD/nonalcoholic steatohepatitis patients were included. We calculated pooled effect upon a meta-analysis, determined correlations (between transaminases and characteristics of patients/exercise) by meta-regression, and assessed the influencing variable through subgroup analysis.

**Results:** A total of 18 studies (22 trials) with 1098 NAFLD patients (exercise = 568; control = 530) were included. The pooled outcomes revealed that exercise intervention significantly decreased both ALT (*p* = 0.004) and AST (*p* = 0.001) levels in NAFLD patients. Meta-regression analysis showed decreased ALT (coef. = 1.138, *p <* 0.01) and AST (coef. = 0.459, *p* = 0.041) after intervention was correlated with the age of patients. Particularly, patients aged 30–39 years (MD: −25.89 U/L, 95% CI: −36.40 to −15.37, *p <* 0.00001) and 40–49 years (MD: −12.17 U/L, 95% CI: −20.38 to −3.96, *p* = 0.004) represented a substantial decrease in ALT levels. Additionally, the 50–59 years age group tended to have decreased ALT levels (MD: −3.94 U/L, 95% CI: −8.19 to 0.31, *p* = 0.07); however, patients above 60 years did not respond (*p* = 0.92) to exercise intervention. In contrast, exercise-induced AST reduction was found in only the 30–39 years age group (MD: −11.92 U/L, 95% CI: −16.78 to −7.06, *p* < 0.00001) and not in patients under the 40–49 (*p* = 0.19), and 50–59 groups (*p* = 0.12) and above 60 years (*p* = 0.15).

**Conclusion:** Our findings suggest that the age of NAFLD patients may be an important variable in improving the levels of serum transaminases, and clinically young patients may have greater benefits from exercise than older patients.

## Introduction

Nonalcoholic fatty liver disease (NAFLD) is a component of metabolic syndrome which is characterized by excessive accumulation of hepatic fat (≥5%) and is not caused by excessive consumption of alcohol, overuse of hepato-toxic medications, or other chronic liver disorders (Friedman et al., 2018). NAFLD constitutes a spectrum of liver disorders that gradually progress from simple steatosis to nonalcoholic steatosis (NASH), fibrosis, cirrhosis, and liver cancer (Bhala et al., 2011). The global prevalence of NAFLD is projected to increase from 25 to 33.5%, and the number of patients in the United States is expected to reach 100 million by 2030 (Estes et al., 2018; Younossi et al., 2018). Clinically, NAFLD is associated with several metabolic comorbidities, including obesity, hyperlipidemia, insulin resistance, type 2 diabetes, and hypertension (Younossi et al., 2016). The existence of these comorbidities further exacerbates the disease burden, and patients with severe NAFLD remain at a high risk of cardiovascular diseases (CVDs) (Targher et al., 2016). The prevalence of NAFLD is higher among older adults with obesity and diabetes; thus, an aging population may suffer severely from the disease (Estes et al., 2018). NAFLD patients with evidence of NASH and advanced fibrosis are highly susceptible to adverse outcomes, including overall mortality and liver-specific morbidity and mortality (Cotter and Rinella, 2020).

NAFLD patients with comorbidities are susceptible to the infection and severity of the coronavirus disease 2019 (COVID-19), during the ongoing global pandemic (Portincasa et al., 2020). A cross-sectional analysis from the US demonstrated that age (>75 years) and body mass index (BMI, >40 kg/m^2^) of COVID-19 patients are strong predictors of hospitalization (Petrilli et al., 2020). A retrospective study from China demonstrated that NAFLD patients had a greater risk of COVID-19 progression, more likelihood of abnormal liver function from admission to discharge, and longer viral shedding time than patients without NAFLD (Ji et al., 2020). Nevertheless, no efficient medical therapy has been endorsed yet for NAFLD treatment. Therefore, to mitigate the NAFLD pathology, effective strategies are necessary for slowing down the disease progression, especially in older patients.

The American Association for the Study of Liver Diseases (AASLD) and other studies have indicated that lifestyle changes, including exercise and dietary interventions associated with weight loss, could be an integral part of NAFLD treatment (Chalasani et al., 2012; Abenavoli et al., 2018). For instance, a higher level of habitual physical activity (PA) is associated with lower intrahepatic fat content, which suggests an inverse correlation between PA and NAFLD outcomes (Perseghin et al., 2007). A clinical trial showed that both aerobic and resistance exercise trainings (8 weeks) equally reduced hepatic fat content and the levels of liver function biomarkers, alanine aminotransferase (ALT) and aspartate aminotransferase (AST) in NAFLD patients. However, the benefits of aerobic exercise were independent of weight loss and decreasing BMI (Shamsoddini et al., 2015). Contrarily, resistance exercise (8 weeks) reportedly reduced liver fat content, promoted fat oxidation, and improved glucose control independent of weight loss in NAFLD patients (Hallsworth et al., 2011). A recent study showed weight-loss-independent benefits of exercise represented by reduced liver steatosis, stiffness, and levels of liver enzymes (AST and ALT) in Japanese men with NAFLD (Oh et al., 2021).

Although either type of exercise has been documented to alleviate NAFLD complications, the degree of exercise benefits may be attributed to the characteristics of patients (age, BMI, and sex differences) or exercise variables (frequency, intensity, duration). In this context, a meta-analysis of randomized controlled trials (RCTs) demonstrated that either type of PA significantly reduced intrahepatic lipid content and blood ALT and AST levels in NAFLD patients. Specifically, patients with high BMI at baseline were represented by an intense decline of liver fat content, while exercise effects were not modified by intensity variables (Orci et al., 2016). Another meta-analysis concluded that decreased intrahepatic triglyceride content after exercise is independent of weight change, but exercise benefits are greater when weight loss occurs in NAFLD patients (Sargeant et al., 2018). A recent meta-analysis of 11 articles reported improved ALT and AST levels, and decreased BMI after long-term exercise in Chinese NAFLD patients, whereas the effects of patient characteristics or exercise variables remain inconclusive (Gao et al., 2021).

So far, no meta-analysis revealed the effect of an exercise intervention on serum transaminase (ALT/AST) levels in NAFLD patients of different age groups. In this study, we conducted a systematic review and meta-analysis of RCTs, and explored the effect of an exercise intervention on transaminases in adult NAFLD/NASH patients. Meta-regression analysis was conducted to discover whether changes in transaminase levels are associated with patient characteristics (age, BMI, and sex) or exercise variables (frequency, intensity, duration). Thereon, subgroup analysis was performed to identify the most sensitive age group of patients in response to exercise intervention.

## Materials and Methods

This study was conducted following the latest Preferred Reporting Items for Systematic Reviews and Meta-Analyses (PRISMA) guidelines (Moher et al., 2009; Page et al., 2021).

### Search Processes

We systematically searched electronic databases, including PubMed, Web of Science, EMBASE, ScienceDirect, and Google Scholar from inception to December 2021. The specific keywords, such as “non-alcoholic fatty liver disease” OR “NAFLD” OR “non-alcoholic steatohepatitis” OR “NASH” OR “fatty liver” OR “hepatic steatosis” AND “exercise” OR “physical activity” OR “aerobic exercise” OR “resistance exercise” OR “strength training” along with “randomized controlled trial” OR “RCT” OR “RCTs” were used to search the articles. The detailed search strategy implemented on the PubMed database has been presented as supplementary data (Supplementary Table S1). In addition, we manually searched the lists of references to obtain other suitable articles.

### Inclusion and Exclusion Criteria

**FIGURE 1:**
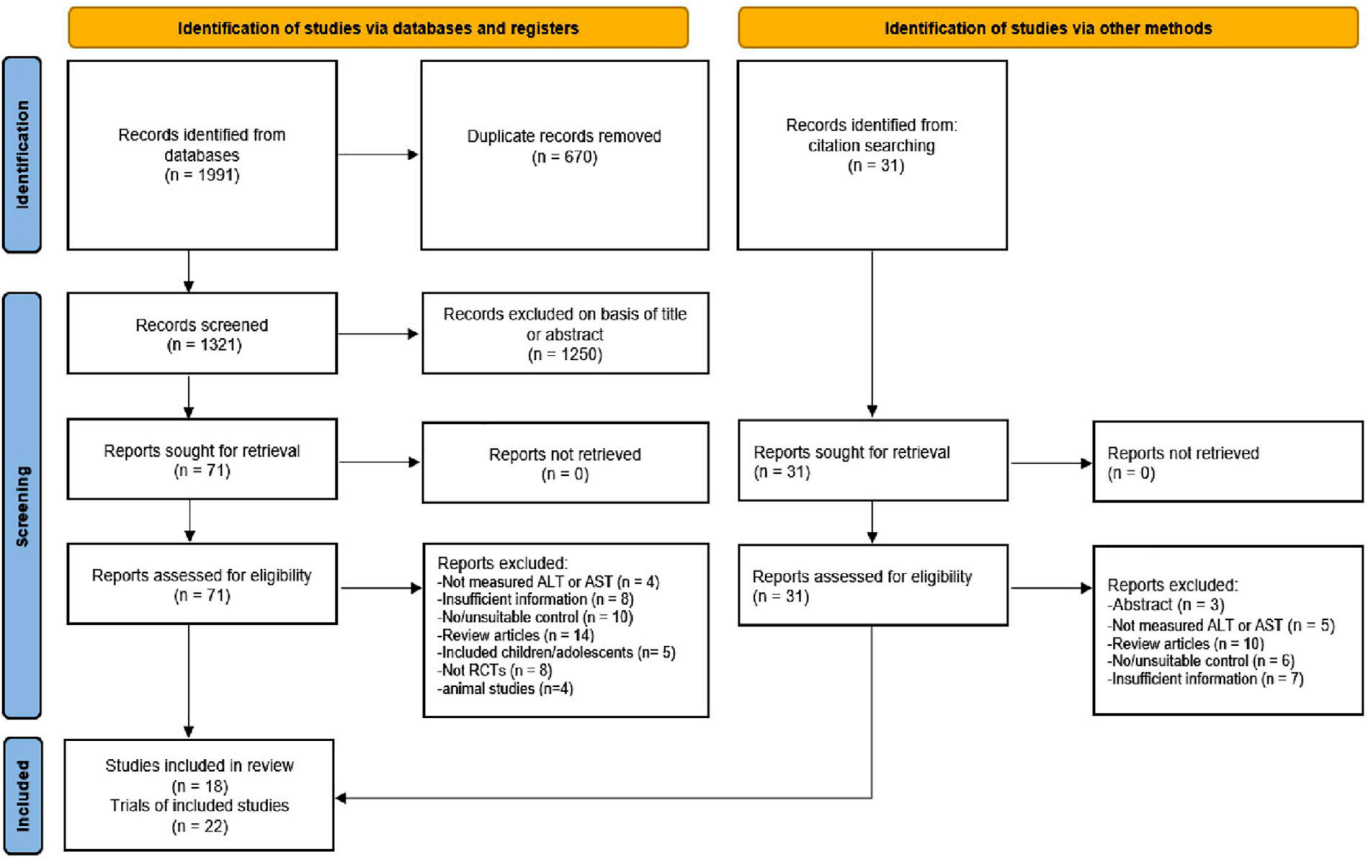
Flowchart of the study selection according to the latest Preferred Reporting Items for Systematic Review and Meta-Analysis (PRISMA).

The inclusion criteria were: 1) All participants in the trials were adults, diagnosed with NAFLD or NASH; 2) the studies should be RCTs with parallel-groups; 3) the intervention group had undergone any type of exercise intervention (such as aerobic, strength, or mobility exercises), whilst the control group had not undergone any exercise intervention; 4) the duration of exercise was 4 weeks or more; and 5) ALT and/or AST levels were assessed per- and post-intervention. The exclusion criteria were: 1) studies conducted on animals; 2) insufficient information of participants (number, age, and BMI), exercise variables (frequency, intensity, duration), and/or outcome measures, and 3) non-original research articles (protocols, meta-analyses, systematic reviews). Based on the inclusion/exclusion criteria, two authors (FH and YL) independently reviewed and assessed the relevant articles. Initially, the titles and abstracts of the identified articles were screened for relevance. Then, the full-text of particular articles were obtained and carefully assessed for the inclusion criteria. Any disagreements were resolved by discussion with another author (MK) to obtain consensus. The detailed study selection process according to the PRISMA guidelines is described in Figure 1.

### Extraction of the Data

Two authors (FH and YL) independently extracted the data using an Excel spreadsheet, followed by verification by review authors (BC and WY). Further cross-checking and in-depth analyses on data were performed by other three authors (VRL, AM, and WY). From each study, the following were extracted: 1) the first author’s last name, year of publication, and country of publication; 2) details of intervention protocol (frequency, intensity, type of exercise, time/duration); 3) characteristics of participants (number, sex, baseline age, and BMI), and 4) pre- and post-intervention means and standard deviations (SD) of clinical outcomes (ALT and AST levels). The outcome values from eligible studies were reported as mean with SD. Standard errors and 95% confidence interval (CI) provided in the included trials were converted to SD using an equation.

### Quality Assessment for the Included Trials

The quality of the included articles was evaluated according to the Cochrane risk of bias tool (Higgins et al., 2011). The Cochrane risk of bias assessment tool consists of the following items: 1) random sequence generation and allocation concealment (selection bias); 2) blinding of participants/personnel (performance bias); 3) blinding of outcome assessments (detection bias); 4) incomplete outcome data (attrition bias); 5) selective reporting (reporting bias); and 6) other sources of bias. The quality of each domain was rated as “low risk,” “high risk,” or “unclear,” indicated with green (+), red (−), and yellow (?) colors and symbols, respectively. The quality of trials was assessed by two authors (FH and YL), and discrepancies were resolved through discussion with a third reviewer (MK) to reach a consensus.

### Statistical Analysis

Cochrane Collaboration’s Review Manager (RevMan 5.3., Copenhagen, and Denmark) was used to statistically analyze the effect of exercise on clinical outcomes (ALT and AST levels) of the NAFLD/NASH patients. The mean difference (MD) and 95% CI were calculated to decide the magnitude of influence of the interventions on the outcomes. To determine heterogeneity, I^2^ statistics was used, with I^2^ ≥ 50% representing high heterogeneity and I^2^ < 50% representing low heterogeneity. The fixed effect model was used to pool the study results when the heterogeneity was low. The random effect model was used to pool the study results when the heterogeneity was high. Based on the heterogeneity significance (pooled outcomes), we performed a meta-regression analysis to identify the correlation between the characteristics of participants (baseline age, BMI, and sex) or exercise variables (frequency, intensity, duration) and changes in clinical outcomes (ALT and AST levels) using the STATA version 12.0 (StataCorp, College Station, TX, United States). The dynamics in ALT and AST levels after exercise were found to correlate with age, and not with BMI, sex, and exercise frequency, intensity, and duration. Hence, to identify the effective age group, we categorized the trials into four subgroups, including patients under 30–39 years, 40–49 years, 50–59 years, and more than 60 years old groups. Sensitivity analysis was performed by conducting a meta-analysis after removing each study sequentially to ascertain if one study biased the pooled results. If the estimate after deleting a study fell outside the 95% CI of the combined effect, the study was considered to have biased the pooled results. The STATA was further used for constructing funnel plots and performing Egger’s test to examine the potential bias in the included RCTs.

## Results

### Search Results and the Selection of Studies

Through systematic search, we identified a total of 2022 articles, of which 1991 were from electronic databases (PubMed, Web of Science, EMBASE, ScienceDirect, and Google Scholar) and 31 were from other sources (i.e., a manual search of the reference lists of included studies and related reviews). After removing duplicates (670), the 1,321 records were retrieved for further assessment. The titles and abstracts of 1,321 articles were examined for suitability, leading to an exclusion of another 1,250 articles. Then, the remaining 102 (71 + 31) articles were carefully reviewed as per the inclusion/exclusion criteria, and 84 articles were excluded for valid reasons. Among them, nine articles did not report ALT or AST level, 15 articles had insufficient information, 16 reported unsuitable controls, 24 were review articles, five included children or adolescents, eight were not RCTs, four were animal studies, and three were only abstracts. Finally, 18 articles were included in the systematic review. Of these 18 articles, one was a three-armed study (3 trials), and two were two-armed studies (4 trials). Thus, a total of 22 trials were included in the meta-analysis. The stages of the article search and study selection process are depicted in Figure 1.

### Summary of the Included Studies and Characteristics of the Patients

Among the 18 RCTs, seven studies were from England (Hallsworth et al., 2011; Pugh et al., 2013; Pugh et al., 2014; Hallsworth et al., 2015; Shojaee-Moradie et al., 2016; Houghton et al., 2017; Whyte et al., 2020), four from China (Wong et al., 2013; Dong et al., 2016; Cheng et al., 2017; Yao et al., 2018), two from the US (Promrat et al., 2010; Sullivan et al., 2012), two from Iran (Shamsoddini et al., 2015; Nikroo et al., 2017), two from Saudi Arabia (Abd El-Kader et al., 2016; Abdelbasset et al., 2019), and one from Brazil (Rezende et al., 2016). All included RCTs were published between 2010 and 2020. Five studies recruited only males, one recruited only females, eight recruited combinations of both sexes, and in four studies, the ratios were not reported. RCTs can provide the most reliable evidence on the effectiveness of intervention (Evans, 2003). To attain high-quality data, we therefore included RCTs in our meta-analysis.

The 21 trials of 18 studies included a total of 1,098 patients diagnosed with NAFLD/NASH. The exercise intervention and control groups were composed of 568 and 530 patients, respectively. The baseline mean age of patients in the trials ranged from 38.67 to 61.28 years, and their mean baseline BMI was from 25.46 to 37.1 kg/m^2^. For the type of exercise, 11 trials included aerobic exercise, three trials resistance exercise, two trials high-intensity interval training (HIIT), and four trials a combination of aerobic and resistance exercises. The frequency of exercise varied from 2 to 5 times per week, and the duration of exercise was ranged from 8 to 103 weeks. The overview of patients and exercise characteristics are presented in Table 1.

**TABLE 1:** Characteristics of the included articles.

Study	Country	Participants (M/F)		Age (Y)	BMI	Exercise type	Intensity	Frequency (t/wk)	Duration (wk)
		Exercise	Control						
Whyte et al. (2020)	England	15 (15/0)	12 (12/0)	57.4	31.6	AE	40–60% HRR	4–5	16
Abdelbasset et al. (2019)	Saudi Arabia	16 (10/6)	16 (9/7)	54.4	36.3	HIIT	80–85% VO_2max_	3	8
Yao et al. (2018)	China	AE:29 (7/22)	31 (13/18)	AE:61.28	AE:25.46	AE	AE:60–70% HRmax	3	22
		RE:31 (16/15)		RE:55.8	RE26.86	RE	RE:60–70% 1RM		
Cheng et al. (2017)	China	AE:22 (5/17)	CO:18 (4/14)	AE:59	AE:27.3	AE	60–75% VO_2max_	2–3	36.9
		AE + DT:23 (7/16)	DT:22 (6/16)	AE + DT:60	AE + DT:26.4				
Houghton et al. (2017)	England	12 (nr)	12 (nr)	54	33	AE + RE	RPE 14–18	3	12
							(hard–very hard)		
Nikroo et al. (2017)	Iran	12 (12/0)	11 (11/0)	38.67	30.37	AE	55–60% HRR	3	8
Dong et al. (2016)	China	130 (130/0)	139 (130/0)	56.68	26.04	AE	60–80% target heart rate (170-age)	3–4	103
Abd El-Kader et al. (2016)	Saudi Arabia	50 (34/16)	50 (36/14)	50.78	32.35	AE	65–75% HRmax	3	12
Rezende et al. (2016)	Brazil	19 (0/19)	21 (0/21)	56.2	34.1	AE	VAT up to 10% below RCP	2	24
Shojaee-Moradie et al. (2016)	England	15 (15/0)	12 (12/0)	52.4	31.6	AE + RE	40–60% HRR	4–5	16
Hallsworth et al. (2015)	England	11 (nr)	12 (nr)	54	31.5	HIIT	RPE 16–17	3	12
							(very hard)		
Shamsoddini et al. (2015)	Iran	AE: 10 (10/0)	10 (10/0)	AE: 39.7	AE: 28.1	AE	AE: 60–75% HRmax	3	8
		RE: 10 (10/0)		RE: 45.9	RE: 30.6	RE	RE: 50–70% 1RM		
Pugh et al. (2014)	England	13 (7/6)	8 (4/4)	50	30	AE	30–60% HRR	3–5	16
Pugh et al. (2013)	England	6 (nr)	5 (nr)	45	31	AE	30–60% HRR	3–5	16
Wong et al. (2013)	China	77 (41/36)	77 (31/46)	51	25.5	AE + RE	Moderate-intensity	3–5	51
Sullivan et al. (2012)	United States	12 (4/8)	6 (1/5)	48.6	37.1	AE	45–55% VO_2max_	5	16
Hallsworth et al. (2011)	England	11 (nr)	8 (nr)	52	32.3	RE	50–70%1RM	3	8
Promrat et al. (2010)	United States	21 (14/7)	10 (8/2)	48.9	33.9	AE + RE	Moderate-intensity	nr	48

Note: M/F, male/female; Y, years; t/wk, times/week; AE, aerobic exercise; RE, resistance exercise; AE + RE, combination of aerobic and resistance exercises; HIIT, high-intensity interval training; Co., control; DT, diet; nr, not reported; VO2max, maximal oxygen uptake; HRR, heart rate reserve; HRmax, maximal heart rate; 1RM, one-repetition maximum; RPE, ratings of perceived exertion (6–20); VAT, ventilatory anaerobic threshold; RCP, respiratory compensation point.

### Exercise Decreases Both ALT and AST Levels in NAFLD/NASH Patients

**FIGURE 2:**
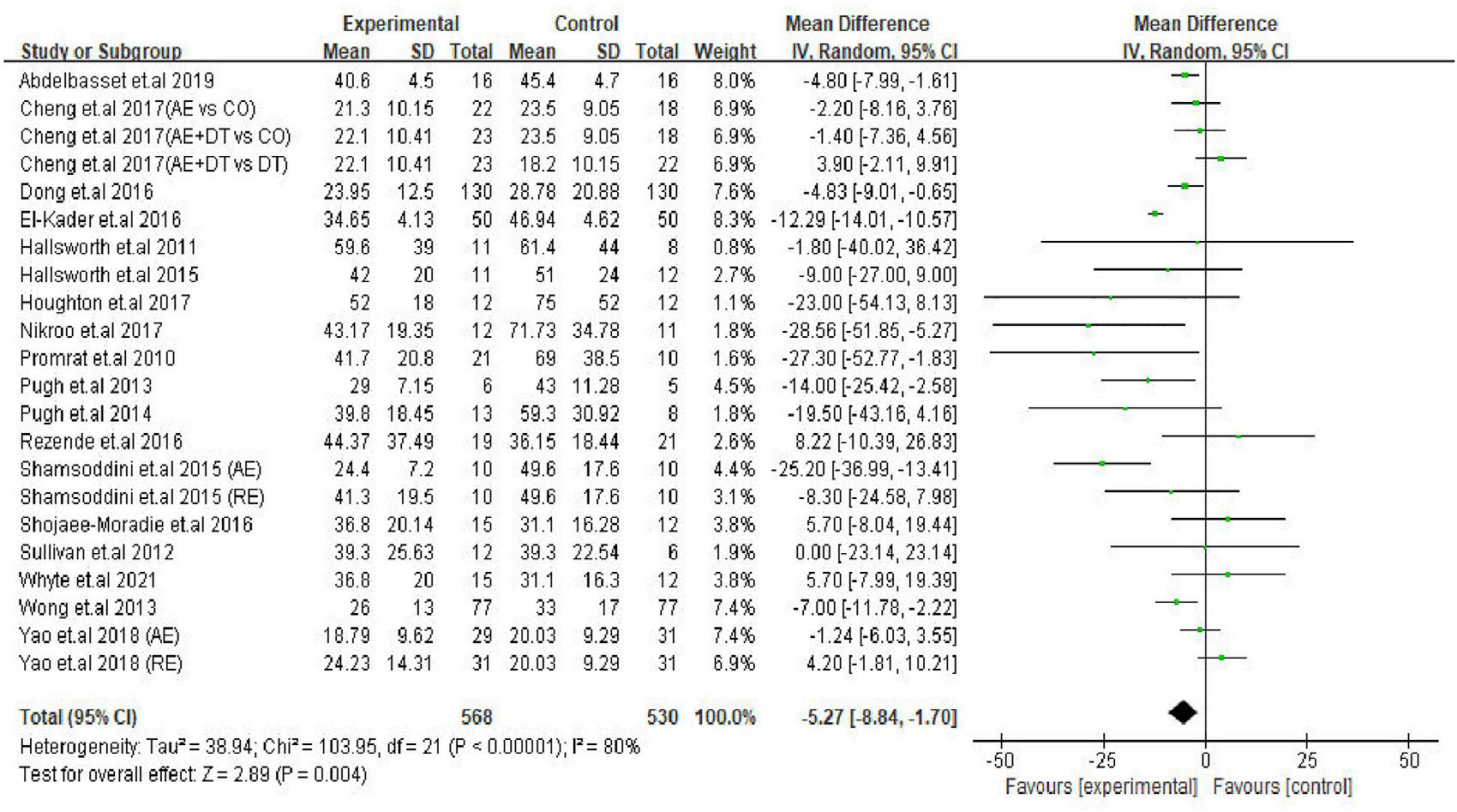
Pooled outcomes of exercise intervention on ALT levels in NAFLD/NASH patients. SD, standard deviation; 95% CI, 95% confidence interval.

**FIGURE 3:**
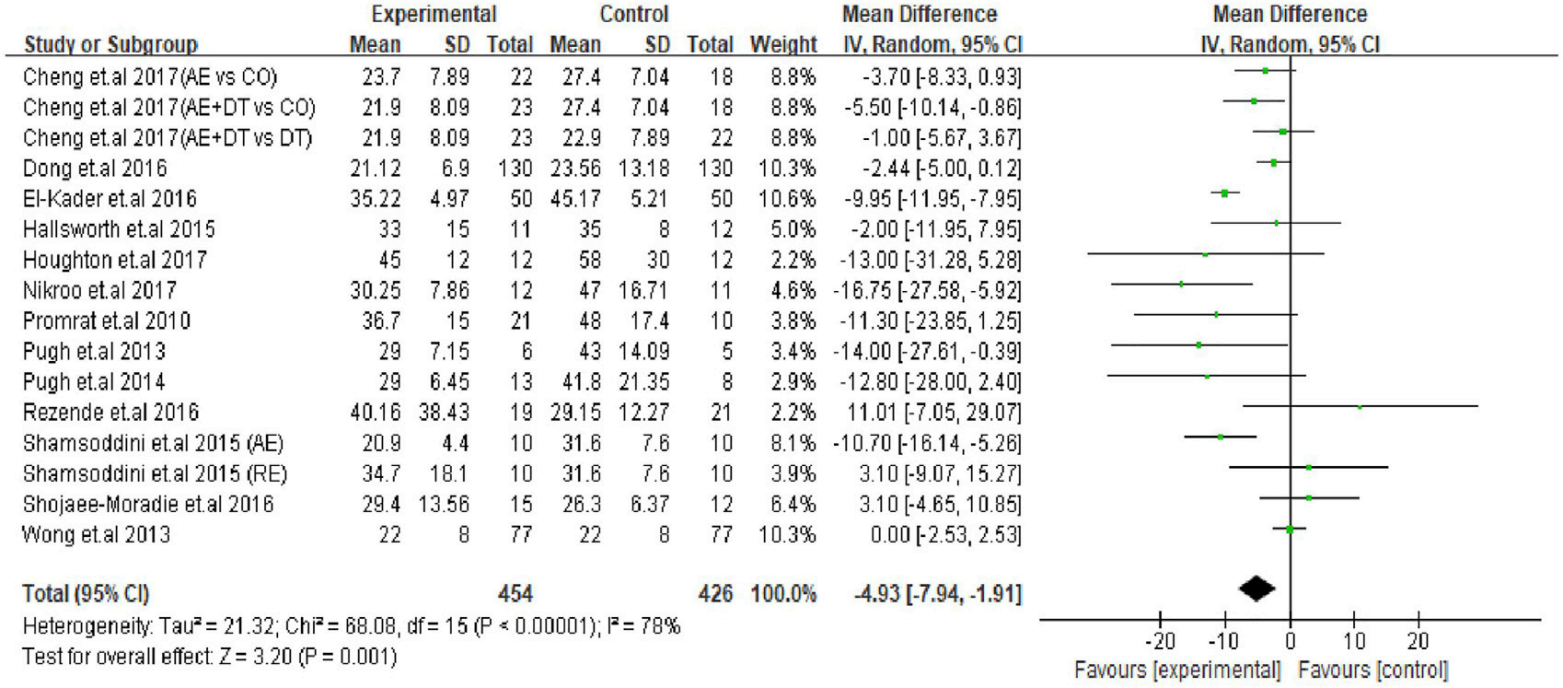
Pooled outcomes of exercise intervention on AST levels in NAFLD/NASH patients. SD, standard deviation; 95% CI, 95% confidence interval.

The effect of an exercise intervention on ALT changes was evaluated in the patients from 22 trials. The pooled results of meta-analysis showed that exercise intervention substantially decreased (*p* = 0.004) ALT levels in patients. The MD in the reduction of ALT levels was -5.27 U/L (95% CI: −8.84 to −1.70), and the heterogeneity was I^2^ = 80% (Figure 2). Next, we identified 16 trials that addressed the effects of exercise on AST levels of NAFLD/NASH patients. Similar to ALT levels, we found exercise intervention significantly decreased (*p* = 0.001) AST levels in patients. The average reduction of AST levels was −4.93 U/L (95% CI: −7.94 to −1.91), and the heterogeneity was I^2^ = 78% (Figure 3). The heterogeneity of both ALT and AST levels indicates that the included trials revealed diverse effects of exercise on ALT and AST changes in adult NAFLD patients.

### Association Between Transaminases and Characteristics of Patients/Exercise Variables

Prevalence of NAFLD is more common among older and obesity adults. Therefore, we performed a meta-regression analysis to determine the source of heterogeneity using age, BMI, and sex variables. We found that the baseline BMI, BMI reduction, and sex differences were not correlated with the reduction of either ALT or AST levels in NAFLD/NASH patients (Table 2). Noteworthy, the ‘age’ of patients significantly correlated with an exercise-induced reduction of ALT (*p* < 0.01) and AST (*p* = 0.041) levels (Table 2). These findings revealed that the beneficial effects of exercise on transaminases are correlated with the “age” of NAFLD patients.

**TABLE 2:** Meta-regression analysis to identify the effective variables.

	Characteristics of patients	Coefficient	Standard error	T-Value	*p*-Value
_**ALT**_	Baseline BMI	−0.4281872	0.5619431	−0.76	0.455
	Age	1.138232	0.2380615	4.78	0.000^a^
	Sex (17 trials)	−10.23261	6.730051	−1.52	0.149
	BMI reduction (19 trials)	−3.137282	2.17553	−1.44	0.167
_**AST**_	Baseline BMI	−0.6276582	0.5291798	−1.19	0.255
	Age	0.459831	0.2045216	2.25	0.041^a^
	Sex (11 trials)	−2.131885	5.426621	−0.39	0.704
	BMI reduction (13 trials)	−2.288405	1.974409	−1.16	0.271

a Represents statistical significance.

Next, we assumed that the exercise characteristics (frequency, intensity, duration) may also be involved in the reduction of ALT or AST levels in NAFLD patients. We performed meta-regression analyses and found that changes in the levels of transaminases were not correlated with exercise frequency (ALT, *p* = 0.872; AST, *p* = 0.147) or exercise intensity (ALT, *p* = 0.076; AST, *p* = 0.325). Similarly, the regression analysis showed that decreased ALT (*p* = 0.508) and AST (*p* = 0.218) levels were not associated with the duration of exercise in NAFLD patients (Supplementary Table S2).

### Exercise Intervention Decreases ALT Levels in Different Age Groups of NAFLD Patients

After finding a significant correlation between age and the levels of transaminases, we categorized the patients of the trials (22) into four age subgroups; including 30–39 years (two trials), 40–49 years (four trials), 50–59 years (13 trials) and ≥60 years old (three trials) groups to identify the most sensitive age group of patients in response to exercise intervention. The prevalence of NAFLD can be seen in adults at the age of early thirties. With advancing age, the risk or severity of the disease would be progressing every 10 years (Kalra et al., 2013; Hu et al., 2018; Tobari and Hashimoto, 2020). A meta-analysis showed a gradual increase of NAFLD incidence in adults of various age groups, including 30–39, 40–49, 50–59 and 60–69 (Younossi et al., 2016). In our study, the average age of patients in trials ranged from 38 to 61 years. Therefore, we assumed that classifying of the patients into four subgroups would be appropriate and imperative to investigate the dynamics of serum transaminases after exercise.

**FIGURE 4:**
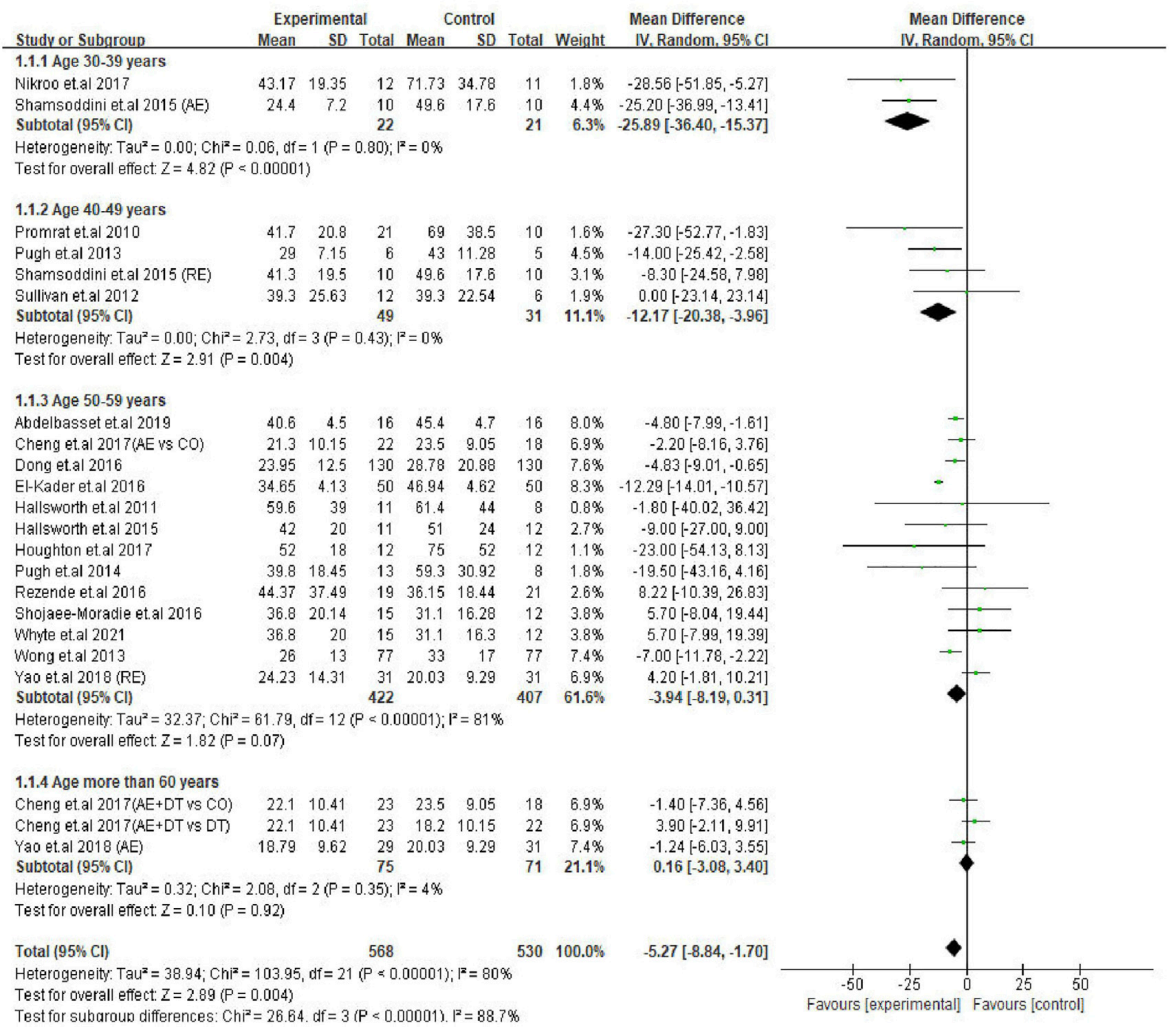
Subgroup analysis of exercise intervention on changes in ALT levels in different age groups of NAFLD/NASH patients. SD, standard deviation; 95% CI, 95% confidence interval.

Subgroup analysis results revealed that young patients were highly responsive to exercise intervention compared with the older, as indicated by improved ALT levels. The decreased ALT level were seen in two age groups, i.e., 30–39 years (MD: −25.89 U/L, 95% CI: −36.40 to −15.37, *p* < 0.00001, I^2^ = 0%) and 40–49 years (MD: −12.17 U/L, 95% CI: −20.38 to −3.96, *p* = 0.004, I^2^ = 0%), whilst the ALT levels in patients in 50–59 years age group tended to decrease with exercise intervention (MD: −3.94 U/L, 95% CI: −8.19 to 0.31, *p* = 0.07, I^2^ = 81%). In contrast, the ALT levels in older adults (≥60 years) were not responsive to exercise intervention (MD: 0.16 U/L, 95% CI: −3.08 to 3.40, *p* = 0.92, I^2^ = 4%). It appears that the effect size of ALT reduction was bigger in younger patients (30–39 years; MD: −25.89 U/L) and gradually lessened as age advanced. Furthermore, the test for subgroup differences showed significant (*p* < 0.00001) differences among the groups (Figure 4).

### Exercise Intervention Decreases AST Levels in Young Patients but Not in Older Patients

**FIGURE 5:**
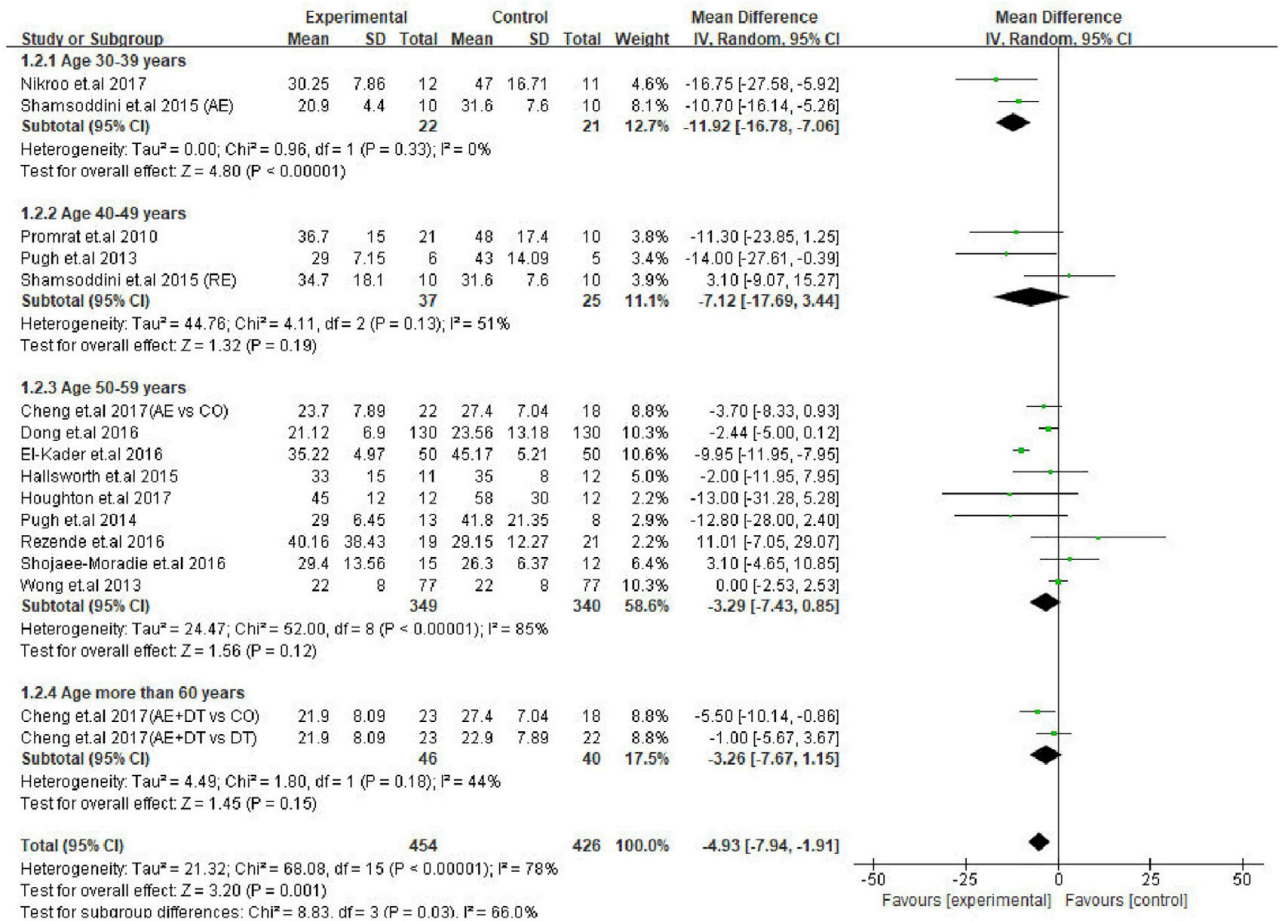
Subgroup analysis of exercise intervention on changes in AST levels in different age groups of NAFLD/NASH patients. SD, standard deviation; 95% CI, 95% confidence interval.

Next, a subgroup analysis was conducted for AST changes to identify the age groups of patients responsive to exercise intervention. The results showed that patients in the 30–39 years age group were represented by a significant decrease in AST levels (MD: −11.92 U/L, 95% CI: −16.78 to −7.06, *p* < 0.00001, I^2^ = 0%). Interestingly, exercise intervention could not alter the AST levels in patients of 40–49 years (MD: −7.12 U/L, 95% CI: −17.69 to 3.44, *p* = 0.19, I^2^ = 51%), 50–59 years (MD: −3.29 U/L, 95% CI: −7.43 to 0.85, *p* = 0.12, I^2^ = 85%) and ≥60 years (MD: −3.26 U/L, 95% CI: −7.67 to 1.15, *p* = 0.15, I^2^ = 44%) age groups. On the other hand, the differences between each subgroup reached statistical significance (*p* = 0.03), which emphasizes the correlation between the age of patients and degree of AST change (Figure 5).

### Summary of Risk of Bias

**FIGURE 6:**
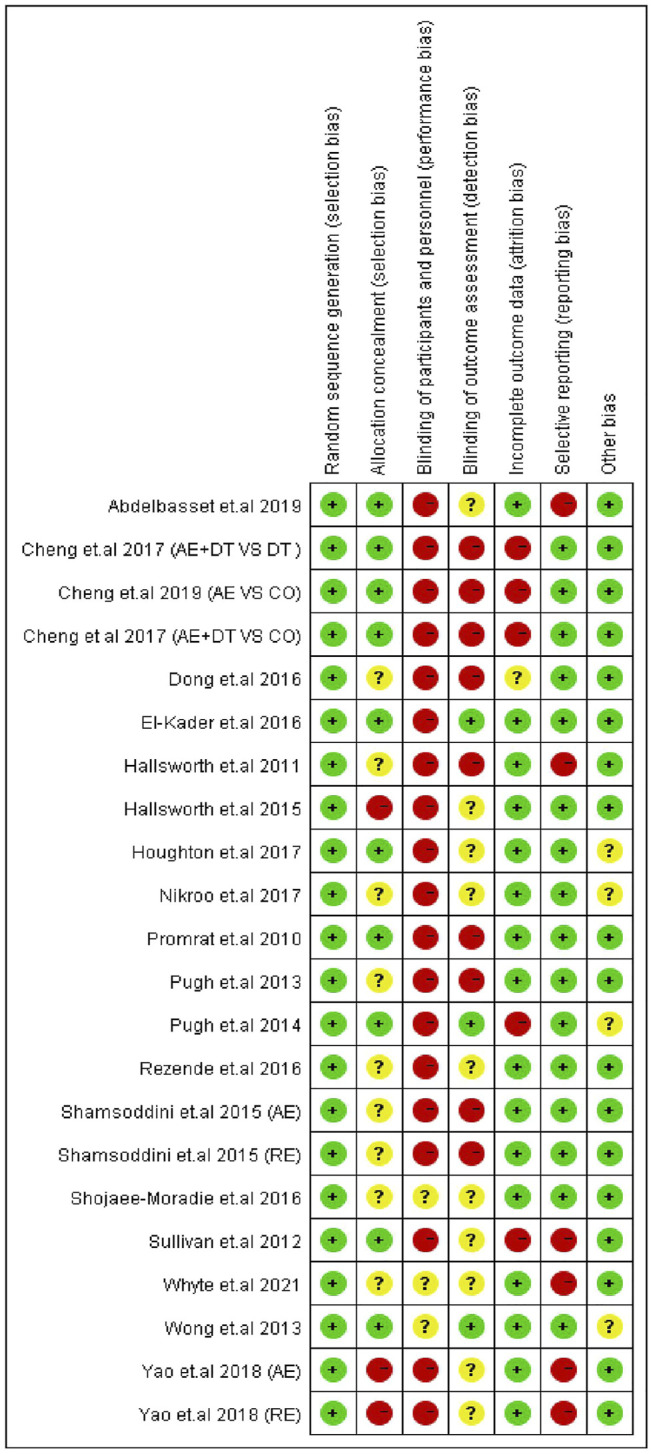
Risk of bias summary of the included studies.

Risk of bias assessment for the 22 trials is presented in Figure 6. For the selection bias, the highest number of studies (22 trials) reported to have low risk of random sequence generation, and ten trials were judged to have a low risk of allocation concealment. As physical exercise was the primary intervention method among all the included trials, it may not have been feasible to adopt the blind method; therefore, several studies were judged as having a high risk of performance bias (19 trials), and a few studies were judged as having a high risk of detection bias (9 trials). However, reporting of such a high risk of performance bias and detection bias does not indicate a compromised quality of the study (Schindhelm et al., 2006; Senior, 2012; Liu et al., 2021). In addition, an attrition bias was identified in five trials, and a reporting bias was identified in six.

We then performed sensitivity analysis by removing each study sequentially to determine if one study biased the pooled results. We found that the pooled results did not vary substantially. The MD upon reduction of ALT levels was from −4.20 (95% CI: −7.32 to −1.09) to −5.90 (95% CI: −9.44 to −2.36), while the MD upon reduction of AST levels was from −4.07 (95% CI: −6.70 to −1.43) to −5.48 (95% CI: −8.52 to −2.44). The sensitivity analysis using STATA software did not show any significant impact on the total effect size of ALT (combined estimate = −5.266, 95% CI: −8.834 to −1.698) and AST (combined estimate = −4.924, 95% CI: −7.937 to −1.91) (Supplementary Figure S1). A funnel plot was produced to ascertain whether significant publication bias was present. Symmetry in the funnel plot shows the absence of publication bias (Supplementary Figure S2). Furthermore, Egger’s test also indicated no publication bias for both ALT (*p* = 0.240) and AST (*p* = 0.929) changes after intervention.

## Discussion

To the best of our knowledge, this is the first systematic review and meta-analysis to explore the exercise-induced benefits on serum transaminases in NAFLD patients among different age groups. Meta-analysis of 18 RCTs (22 trials) revealed that exercise intervention significantly decreased both ALT and AST levels. Through meta-regression analysis, we identified “age” as a key factor that influences the exercise-induced beneficial effects on transaminases. Specifically, exercise caused a significant reduction of ALT levels in patients aged 30–39 and 40–49 years, while a decreased tendency was seen in patients aged 50–59 years. Contrarily, ALT levels in patients aged 60 years and above were not responsive to exercise intervention. On the other hand, exercise-induced reduction of AST levels was noticed only in the 30–39 years age group and not in other age groups (40–49, 50–59, and ≥60 years). Taken together, exercise training is effective in reducing the levels of transaminases in NAFLD patients, irrespective of type, but its efficacy is progressively reduced along with aging.

NAFLD is the most common liver disease, and its incidence rate is continually rising (Huang et al., 2021). This chronic disease does not only lead to advancing stages of liver damage and death, but it is also closely associated with the incidence and/or progression of type 2 diabetes and CVDs (Cotter and Rinella, 2020; Deprince et al., 2020). Typically, NAFLD is a metabolic disorder caused by insulin resistance, which causes too many free fatty acids to enter the liver, and promotes the production and accumulation of total triglycerides (TG) in the liver. Accumulation of a large amount of TG in the liver subsequently leads to the excessive production of pro-inflammatory cytokines and free radicals, which then impairs liver function (Rolo et al., 2012). On one hand, a sedentary lifestyle or physical inactivity is an important reason for the incidence of NAFLD and elevation of levels of liver biomarkers, including transaminases. On the other hand, lifestyle modification or regular PA is beneficial for patients in decreasing the clinical outcomes and disease burden (Chalasani et al., 2012; Golabi et al., 2020). A study from Israel reported that regular PA is associated with a lower prevalence of NAFLD (Zelber-Sagi et al., 2008). A recent longitudinal analysis (10.6 years) identified that longer duration of total PA and moderate-to-vigorous PA are associated with a lower all-cause mortality and cardiovascular mortality in NAFLD patients (Kim et al., 2021). Another RCT from China reported that 6- or 12-months of moderate exercise and vigorous-moderate exercise effectively decreased intrahepatic TG contents in NAFLD patients (Zhang et al., 2016). In insulin-resistant individuals, exercise training has been shown to improve skeletal muscle glycogen synthesis with a concomitant decrease of *de novo* lipogenesis and hepatic TG synthesis (Rabøl et al., 2011). It has been claimed that exercise can reduce hepatic fat or TG content in patients through improved insulin resistance, fatty acid oxidation, and mitochondrial function and the activation of inflammatory or antioxidant cascades (van der Windt et al., 2018)

Both transaminases, ALT and AST, are commonly used as biomarkers to determine the physiological status and function of the liver. The ALT is primarily produced in the cytoplasm of hepatocytes, while AST is mainly produced in the mitochondria of hepatocytes. Upon hepatocyte damage, the permeability of cell membrane increases, and ALT is released into the blood. If hepatocytes are seriously damaged or persist, the mitochondria in hepatocytes will be damaged, and AST will be released into the blood (Schindhelm et al., 2006; Senior, 2012). Previous studies have focused on the effect of exercise on transaminases in NAFLD patients, but the results are equivocal. For instance, 8 weeks of aerobic exercise and resistance training were equally effective in reducing the ALT and AST levels as well as hepatic fat content in the NAFLD patients, but the efficiency of aerobic exercise was independent of weight loss (Shamsoddini et al., 2015). Another study showed that resistance training (8 weeks) decreased liver fat and improved fat oxidation in NAFLD patients without affecting body weight or whole-body fat mass (Hallsworth et al., 2011). Fealy et al. reported a significant decrease in ALT levels, but not AST levels and intrahepatic fat contents, in NAFLD patients after 7 days of treadmill exercise (Fealy et al., 2012). The main reasons for this discrepancy may be factors, including characteristics of patients (i.e., age, BMI, and sex) or exercise intervention (i.e., frequency, intensity, and duration).

The pooled outcomes of our meta-analysis of 22 RCTs showed exercise intervention to significantly decrease both ALT and AST levels in NAFLD patients. A previous meta-analysis reported an exercise-induced decrease of transaminase levels in NAFLD patients, and such decrease correlated with the baseline BMI of patients (Orci et al., 2016). Conversely, another meta-analysis of 10 trials concluded that either exercise intervention alone or in combination with diet had no effect on ALT levels in NAFLD patients (Keating et al., 2012). In a recent meta-analysis, Wang et al. categorized the included trials into two groups based on intervention duration and found that the longer duration of exercise (≥4 months) tended to decrease the ALT and AST levels in NAFLD patients, while the shorter duration (<4 months) had no effect (Wang et al., 2020). A network meta-analysis revealed aerobic exercise to be effective in improving the ALT and AST activities compared with progressive resistance exercise in NAFLD patients (Zou et al., 2018). These previous meta-analyses clearly demonstrated that factors including the bodyweight of patients, type of exercise, and duration of exercise are involved in controlling AST and ALT levels in NAFLD patients. Nevertheless, the influence of “age” on exercise-induced alternations of ALT and AST levels remains inconclusive in NAFLD patients.

Identification of the variables that are critically involved in the manifestation of liver pathology is vital in the treatment of NAFLD. Literature revealed that the prevalence of NAFLD can be seen in adults at the age of early thirties, and the disease burden would be increased for every 10 years (Kalra et al., 2013; Hu et al., 2018; Tobari and Hashimoto, 2020). In this context, a meta-analysis reported progressively increased incidence of NAFLD in adults of various age groups, such as 30–39 (22.43%), 40–49 (26.53%), 50–59 (27.40%), and 60–69 (28.90%) years (Younossi et al., 2016). These findings emphasize that the prevalence of NAFLD is increasing in adults for every 10 years of age from 30 to 69 years. In our study, we found “age” of patients to significantly correlate with the exercise-induced reduction of ALT and AST levels in patients but not the BMI of patients. Thus far, there are no reports which delineate the role of the age of patients on exercise-induced improvements of transaminase levels; thus our findings signify the clinical vitality of “age” in the treatment of NAFLD. We then classified the trials into four age subgroups based on a previous study (Younossi et al., 2016). We further emphasized that compared to older patients, younger patients were highly responsive to exercise intervention in the improvement of transaminases. This was evidenced by a substantial decrease in ALT levels in both 30–39 to and 40–49 years age groups and a substantial decrease in AST levels in 30–39 years age group of patients.

With increasing age, the structure and function of liver change considerably in older NAFLD patients, and that is related to the significant damage of hepatic metabolism and detoxification (Bertolotti et al., 2014). A study from Japan found age to be significantly associated with the development of NASH in male NAFLD patients and progression of fibrosis in female NASH patients (Shima et al., 2015). Similarly, a prospective study conducted on 44 NAFLD patients (mean age 36.5 years) showed significantly decreased ALT and AST levels after exercise (Baba et al., 2006). In another RCT, NAFLD patients aged 44.16 years showed decreased ALT and AST levels after aerobic exercise (Draz et al., 2020). In contrast, older NAFLD patients with pre-diabetes were unable to revert their transaminases even after 8 months of exercise combined with dietary intervention (Cheng et al., 2017). These findings reveal the importance of age in attenuating the levels of liver function biomarkers and pathology. It is key to note that younger people generally play an important role in social and economic development. However, owing to busy schedules, PA of young individuals may decline, which results in a large amount of fat accumulation. Excessive fat accumulation in the liver eventually causes inflammation and oxidative injury to hepatocytes and decreases liver function. Therefore, decreasing transaminase levels in young or older NAFLD patients is necessary to prevent and/or reverse the liver damage.

## Limitations

The included articles in this study were obtained through a comprehensive systematic search of major electronic databases and manual search. However, the records identified from grey literature sources did not fulfill our inclusion criteria for meta-analysis. Although some adult NAFLD patients would invariably have used drugs along with exercise intervention to control the clinical manifestations of the disease, we did not consider or adjust the drug effect on reported outcomes. Furthermore, two trials were included more than once in our analysis, and this may have promoted bias in the results. In the subgroup analysis, the relative weights of four subgroups appeared to be different from each other. Such differences in weights may be due to the number of trials and/or number of patients included in certain age groups. Although exercise has significant effect on transaminases, the small effect size in this study may not strongly convey the practical application of intervention in the clinical management of NAFLD. Further analysis with an adequate number of studies and a precise classification of age subgroups may be warranted to evaluate the intervention effect exercise on transaminases.

## Conclusion

The evidence from our systematic review and meta-analysis showed age to be one of the key variables in improving the serum transaminase levels of NAFLD patients. Specifically, exercise can decrease both ALT and AST levels in young NAFLD patients, while with aging, this efficacy was progressively reduced (in older patients). Therefore, NAFLD patients should undergo adequate exercise sessions as soon as possible upon diagnosis of the disease.

## Data Availability

The original contributions presented in the study are included in the article/Supplementary Material, further inquiries can be directed to the corresponding authors.
