# Supplementary material for: Effect of Exercise Training on Serum Transaminases in Patients With Nonalcoholic Fatty Liver Disease: A Systematic Review and Meta-Analysis
Source: Front Physiol. 2022 Jun 28;13:894044. doi: 10.3389/fphys.2022.894044 (PMC9273783; doi:10.3389/fphys.2022.894044)

**Effect of Exercise Training on Serum Transaminases in Patients with Nonalcoholic Fatty Liver Disease: A Systematic Review and Meta-Analysis**

Feng Hong^1#^, Yubo Liu^2,3#^, Veeranjaneya Reddy Lebaka^4^, Arifullah Mohammed^5^, Weibing Ye^2^, Biqing Chen^1*^ and Mallikarjuna Korivi^2*^

**Supplementary data**

**Supplementary Table 1S. Search strategy to identify the relevant articles on PubMed.**

| **PubMed:** | ((((((((((Non-alcoholic Fatty Liver Disease[Title/Abstract]) OR (Non alcoholic Fatty Liver Disease[Title/Abstract])) OR (NAFLD[Title/Abstract])) OR (Nonalcoholic Fatty Liver Disease[Title/Abstract])) OR (Fatty Liver*, Nonalcoholic[Title/Abstract])) OR (Liver*, Nonalcoholic Fatty[Title/Abstract])) OR (Nonalcoholic Fatty Liver*[Title/Abstract])) OR (Nonalcoholic Steatohepatitis*[Title/Abstract])) OR (Steatohepatitis*, Nonalcoholic[Title/Abstract])) AND (((((((((((((((Exercise[Title/Abstract]) ) OR (Exercise*[Title/Abstract])) OR (Physical Activity*[Title/Abstract])) OR (Activity*, Physical[Title/Abstract])) OR (Exercise*, Physical[Title/Abstract])) OR (Physical Exercise*[Title/Abstract])) OR (Acute Exercise*[Title/Abstract])) OR (Exercise*, Acute[Title/Abstract])) OR (Exercise*, Isometric[Title/Abstract])) OR (Isometric Exercise*[Title/Abstract])) OR (Exercise*, Aerobic[Title/Abstract])) OR (Aerobic Exercise*[Title/Abstract])) OR (Exercise Training*[Title/Abstract])) OR (Training*, Exercise[Title/Abstract]))) AND ((((Randomized controlled trial[Title/Abstract]) OR (Randomized[Title/Abstract])) OR (RCT[Title/Abstract])) OR (RCTs[Title/Abstract])) |
| --- | --- |

We have followed the above search strategy to find the relevant articles from the PubMed database.

**Search Results:**

- We found a total of 209 studies that matches to the given keywords.
- After screening and reviewing the full-text articles, 8 studies were included.

**List of records included in the systematic review and meta-analysis:**

1. Abdelbasset et.al 2019
2. Cheng et al. 2017
3. Dong et.al 2016
4. Rezende et.al 2016
5. Shojaee-Moradie et al. 2016
6. Pugh et.al 2013
7. Pugh et.al 2014
8. Sullivan et.al 2012.

**Supplementary Table 2S. Meta-regression analysis for exercise frequency, intensity and duration.**

|  | **Exercise characteristics** | **Coefficient** | **Standard Error** | **T-Value** | **p-Value** |
| --- | --- | --- | --- | --- | --- |
| **ALT** | Frequency (12 trials) | -1.050597 | 6.366631 | -0.17 | 0.872 |
|  | Intensity | -7.178571 | 3.835437 | -1.87 | 0.076 |
|  | Duration | 0.0550534 | 0.0817532 | 0.67 | 0.508 |
| **AST** | Frequency (7 trials) | -20.71737 | 12.06776 | -1.72 | 0.147 |
|  | Intensity | -3.320546 | 3.252965 | -1.02 | 0.325 |
|  | Duration | 0.0671536 | 0.0521011 | 1.29 | 0.218 |

Changes in ALT and AST were not significantly associated with exercise frequency, intensity or duration.

**Supplementary Figure 1S. Sensitivity analysis of included trials for both ALT and AST.**

**ALT**


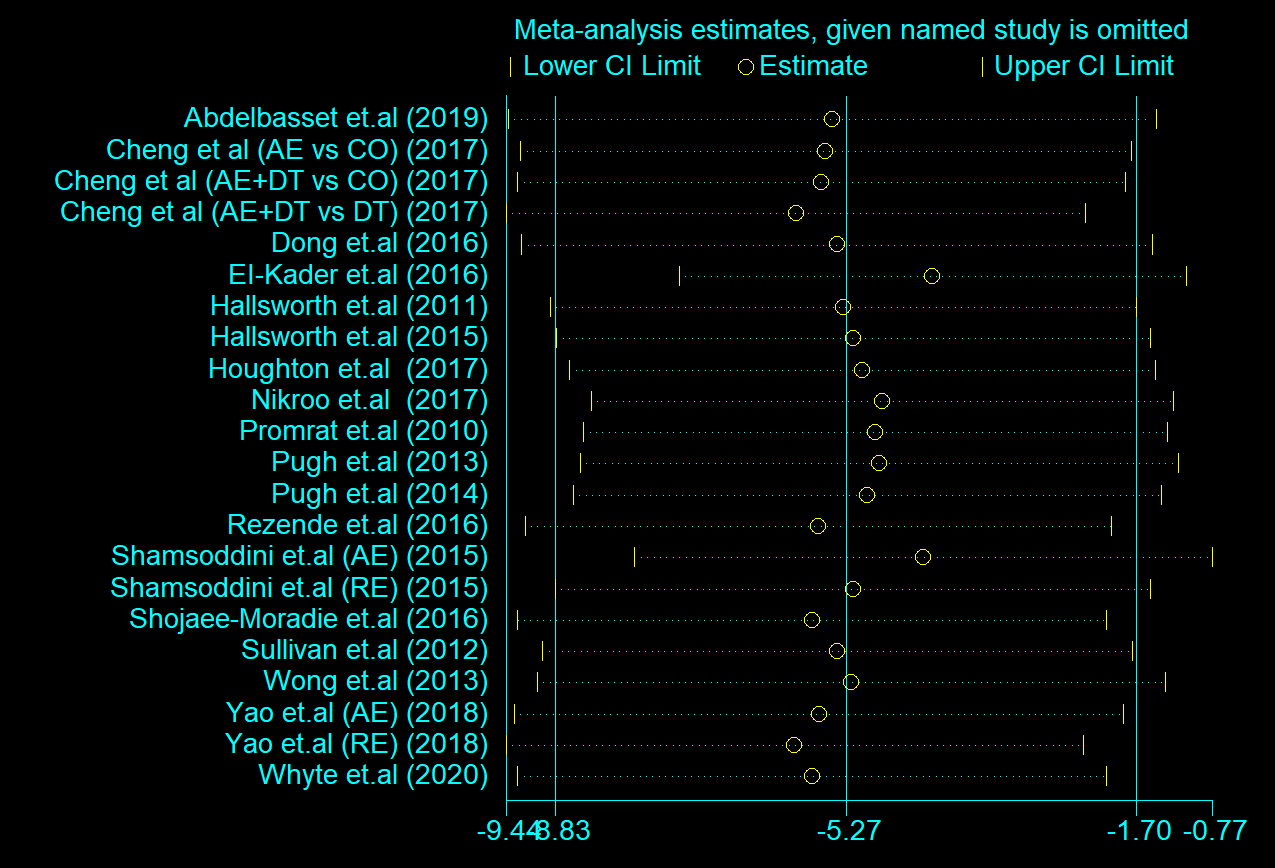


**AST**


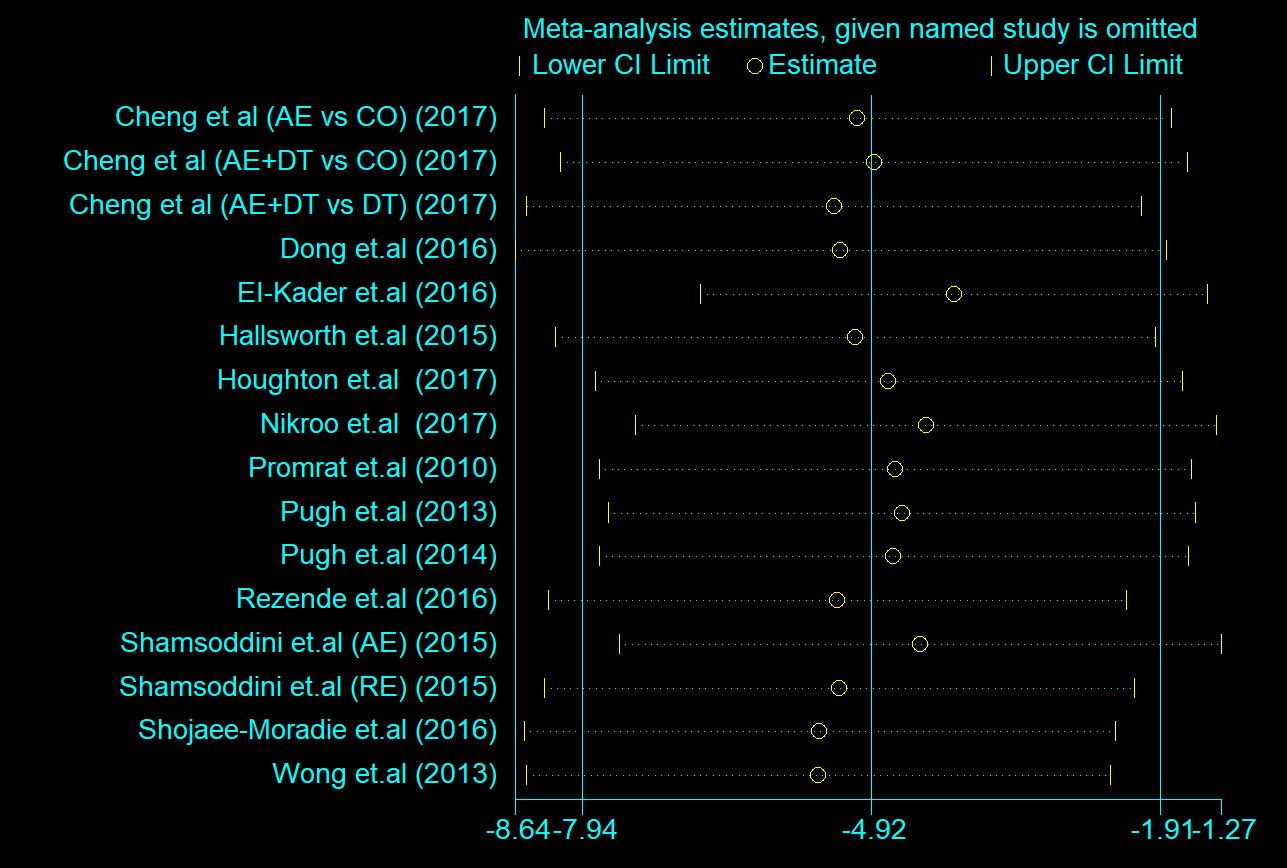


**Supplementary Figure 2S. Funnel plot results for the included studies.**

**ALT**


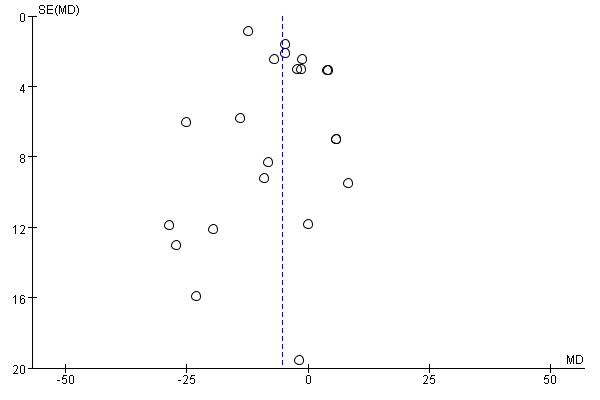


**AST**


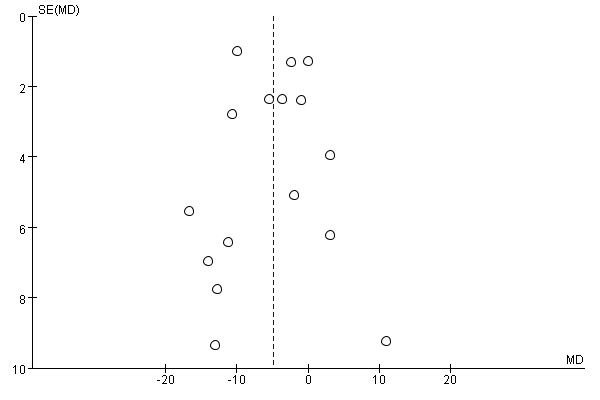

Supplement: Supplementary file 1 [file Table1.DOCX]
